# Multidisciplinary Approach to Perihilar and Intrahepatic Cholangiocarcinoma: A Single-Center Experience

**DOI:** 10.5152/tjg.2024.24301

**Published:** 2025-01-01

**Authors:** Adnan Gundogdu, Taha Anil Kodalak, Hakan Kucukaslan, Serdar Topaloglu, Ummuhan Turali, Sukru Oguz, Davut Dohman, Hasan Dinc, Feyyaz Ozdemir, Mehmet Arslan, Umit Cobanoglu, Erdem Karabulut, Adnan Calik, Mehmet Halil Ozturk

**Affiliations:** 1 Department of Surgery, Karadeniz Technical University School of Medicine, Trabzon, Türkiye; 2 Department of Pathology, Karadeniz Technical University School of Medicine, Trabzon, Türkiye; 3 Department of Radiology, Karadeniz Technical University School of Medicine, Trabzon, Türkiye; 4 Department of Anesthesiology, Karadeniz Technical University School of Medicine, Trabzon, Türkiye; 5 Department of Medical Oncology, Karadeniz Technical University School of Medicine, Trabzon, Türkiye; 6 Department of Gastroenterology, Karadeniz Technical University School of Medicine, Trabzon, Türkiye; 7 Department of Biostatistics, Hacettepe University, Ankara, Türkiye; 8 Department of Radiology, Sakarya University Faculty of Medicine, Sakarya, Türkiye

**Keywords:** Intrahepatic cholangiocarcinoma, perihilar cholangiocarcinoma, postoperative outcome, prognostic factors, surgical treatment

## Abstract

**Background/Aims:**

Treatment of perihilar cholangiocarcinoma (PHCC) and intrahepatic cholangiocarcinoma (IHCC) is a challenging issue. We aimed to investigate the clinical characteristics of both tumors and the outcome of our treatment policy.

**Material and Methods:**

We retrospectively analyzed data of 117 patients who were diagnosed with PHCC or IHCC between January 2007 and September 2023. Postoperative outcomes and the effects of prognostic factors on overall survival (OS) were investigated.

**Results:**

Surgical resection was performed on 47 patients (PHCC, n = 33 and IHCC, n = 14). Preoperative biliary drainage was applied in 32 of 33 cases with PHCC and 2 of 14 cases with IHCC. The mortality rate was 8.5% (n = 4). The complication rate was 68.1%. The R0 resection rate was 73% in PHCC. The mean OS time of PHCC cases that underwent R0 resection was 26.5 ± 24.8 months. The mean OS time of patients who underwent resection for IHCC was 28.7 ± 35.5 months. The OS was poorly affected by high CA19-9 levels (≥37 U/mL) (*P* = .005), the presence of lymphovascular invasion (*P* = .049), positive surgical margins after resection (*P* < .001), and the development of postoperative acute renal failure (*P* = .078). The OS of patients receiving adjuvant chemotherapy was significantly longer (*P* = .071). CA19-9 levels of more than 37 U/mL (*P* = .027) and positive surgical margin (*P* < .001) were independent factors for poor OS.

**Conclusion:**

Surgical resection is the mainstay of multidisciplinary treatment for PHCC and IHCC. In advanced stages of IHCC, the combination of loco-regional therapies and repeat surgery, along with the enhanced efficacy of systemic chemotherapy, plays a significant role in a patient’s survival.

**Main Points**

- Surgical resection is the mainstay of multidisciplinary treatment for perihilar cholangiocarcinoma (PHCC) and intrahepatic cholangiocarcinoma (IHCC).
- Our research pointed out the prognostic significance of CA 19-9 level, surgical margins after resection, lymphovascular invasion, postoperative renal failure, and adjuvant chemotherapy among PHCC and IHCC patients.
- In advanced stages of IHCC, the combination of loco-regional therapy and repeat surgery, along with the enhanced efficacy of systemic chemotherapy, plays a significant role in a patient’s survival.

## Introduction

Cholangiocarcinoma (CC) is a rare but intractable malignancy because of the high rate of advanced or metastatic disease at the initial presentation.^1^ The majority of risk factors for the onset of CC are associated with long-term irritation and inflammation.^1^ Liver resection is a mandatory part of the surgical treatment of perihilar CC (PHCC) or intrahepatic CC (IHCC). The principal objective of surgical management is reduced post-operative mortality and a complete (R0) resection. The 5-year overall survival (OS) rate is about 30% in large series.^1^ The prognostic factors, including serum carbohydrate antigen (CA) 19-9 level, lymph node status, resection margin status, pathological differentiation, the use of adjuvant therapy, and the presence of perineural invasion, have been investigated in the literature.^1-3^

We summarize 16 years of institute’s experience in diagnosis and management of PHCC and IHCC, with emphasis on surgical outcomes. The study was also aimed to determine the prognostic factors for achieving an optimal treatment strategy.

## Materials and Methods

Patients who were diagnosed with PHCC or IHCC between January 2007 and September 2023 were included in the study. The Ethics Committee of Karadeniz Technical University approved this retrospective study (Approval number: 2023/223, date: 22 November 2023). Informed consents of the patients have been received before treatment.

Contrast-enhanced triphasic computed tomography (CT) and magnetic resonance cholangiopancreatography (MRCP) were used as initial diagnostic tools. Massive involvement of the main trunk of the portal vein and/or hepatic artery and/or extrahepatic metastases precluded resection in patients with PHCC. The volume (≥40%) and function of the future remnant liver were also considered for the decision of tumor resection. If patients with obstructive jaundice were considered for tumor resection, preoperative percutaneous transhepatic biliary drainage (PTBD) of at least the future liver remnant was performed. We did not perform a preoperative tumor biopsy or intraluminal brushings. We performed radical resection after complete recovery from jaundice, i.e., a total bilirubin decrease to 2.0 mg/dL.^3^ With the help of CT, MRCP, and PTBD images, PHCCs were classified according to Bismuth–Corlette.^4^ The hypo-vascular finding of the mass on imaging confirms the diagnosis of IHCC. Gastrointestinal metastases are also excluded.^1^ We did not perform a preoperative tumor biopsy. Multiple intrahepatic tumors, metastatic disease, and locally progressed solitary tumors impacting either inflow or outflow bilaterally are criteria for unresectability of IHCC.^1^ In patients with normal liver function, resection of up to 80% of the hepatic volume may be considered; in those with impaired liver function, it may be up to 60%.

Extrahepatic bile duct resection (BDR) and complete lymphadenectomy of the hepatoduodenal ligament were performed for *Bismuth-Corlette* type I tumors. Right hepatectomy for *Bismuth-Corlette* type II and type IIIa tumors or left hepatectomy for *Bismuth-Corlette* type IIIb tumors was performed in addition to BDR and lymphadenectomy. Extended right or left hepatectomy was performed for the resection of *Bismuth-Corlette* type IV tumors.^3^ We did not perform routine lymph node dissection in the surgical treatment of IHCC until 2015 (n = 9/14). After the declaration of the expert consensus statement on the topic by Weber et al,^5^ we started to perform routine lymph node dissection in resectable IHCC cases (n = 5/14).

Our surgical technique under low central venous pressure (CVP) was described before.^6-9^ After confirmation of tumor resectability in PHCC patients, the common bile duct was transected distally and liberated from the remnant hepatic artery and portal vein. Prior to resection, the liver was mobilized and the ipsilateral inflow and outflow were controlled extra-parenchymally where possible. Liver transection was performed under intermittent portal triad clamping (PTC) in 19 of 33 patients with PHCC. Portal vein resection (PVR) was undertaken at the final step just before removing the specimen. The portal vein was repaired using 6-0 monofilament non-absorbable sutures (Polypropylene-Prolene, Ethicon, Somerville, NJ, USA). An autologous umbilical vein graft was used for reconstruction of the portal vein defect in the presence of a large portal vein defect. Tumor extension on the distal choledochal margin was examined with a frozen section biopsy. After confirmation of a tumor-negative distal choledochal margin (n = 33/33), the bilioenteric anastomosis was performed in a Roux-en-Y fashion with interrupted 5-0 monofilament absorbable sutures (Polydioxanone-PDS, Ethicon, Somerville, NJ, USA). Liver transection was performed under intermittent PTC in all patients with IHCC.

Antibiotic prophylaxis and prophylaxis for venous thrombosis were applied according to our policy reported previously.^6-9^ Fresh frozen plasma was used to replace hemorrhage that occurred after liver resection (n = 34/47). In 12 out of 47 resections, erythrocyte suspension transfusion was necessary. Of the 47 patients, 44 were successfully extubated in the operating room. Following extubation, patients (n = 40/47) with uneventful operative courses were sent to the surgical ward.

One day following extubation, patients underwent our standardized pulmonary care program.^8^ Postoperative complications were categorized using Dindo’s classification system.^10^ Post-hepatectomy liver failure (PHLF) was determined based on the criteria set forth by the International Study Group of Liver Surgery (ISGLS).^11^ Our algorithm was used to support the failing liver in the instance of PHLF.^12^ Authors used the 8th edition of the American Joint Committee on Cancer (AJCC) staging system for staging of PHCC and the 7th edition of the AJCC staging system for staging of IHCC.^13^

Our PHCC patients in the study group received gemcitabine-based regimens. Intrahepatic CC patients in the study group received 5-FU-based regimens (n = 7/12), gemcitabine (n = 3/12), gemcitabine/cisplatin (n = 1/12), and capecitabine/oxaliplatin (n = 1/12). The treatment options of re-resection of the tumor, transarterial chemoembolization (TACE), or selective internal radiation therapy (SIRT) were also performed for selected cases with recurrent IHCC.^14^

### Statistical Analysis

The data were expressed as median (minimum–maximum) or mean (± SD). The Kaplan–Meier method was used to estimate the disease-free and overall survival, and the log-rank test was used to compare the survival curves. Univariate and multivariate analyses were performed using the Cox proportional hazards regression model. All *P* values were considered statistically significant when the associated probability was less than .05.

## Results

**Figure 1.**
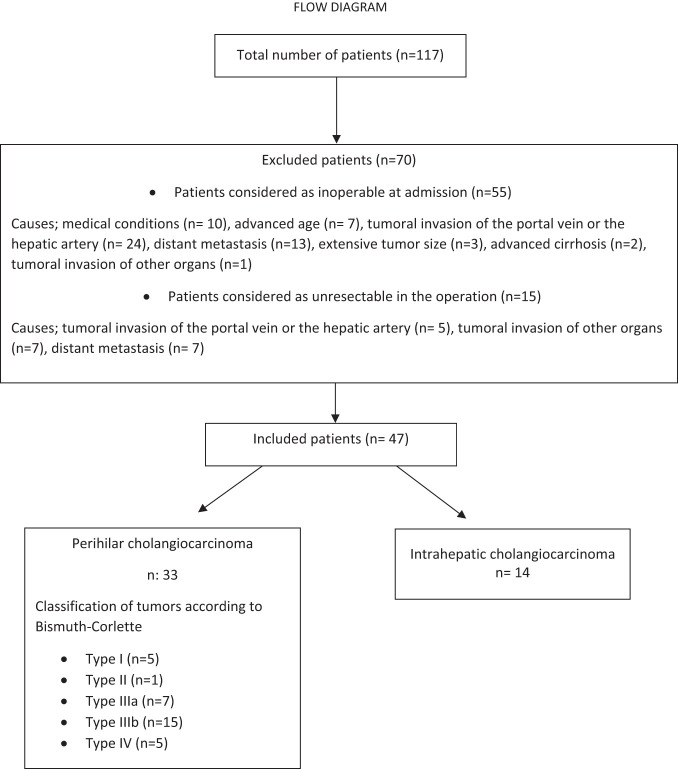
Flowchart of patients who were diagnosed with perihilar or intrahepatic cholangiocarcinoma between 2007 and 2023.

One hundred seventeen patients were admitted for PHCC or IHCC and deemed suitable for inclusion in this study. Of these patients, 55 (47%) were not found suitable for operative treatment. The remaining 62 (52.9%) patients underwent operative treatment, but 15 (24.1%) did not undergo resection. Finally, 47 patients who underwent resection were included in the analysis. The flowchart of patient selection is shown in Figure 1.

The demographics of the patients are summarized in Table 1. Preoperative biliary drainage was applied in 32 of 33 cases with PHCC and 2 of 14 cases with IHCC. Bismuth–Corlette type IIIb was the leading subtype of PHCC in resected cases (n = 15/33). PVR was required in 3 of 33 patients with PHCC. The portal vein was repaired with direct suturing (n = 2) or with an autologous umbilical vein graft (n = 1). The median tumor size of the patients with IHCC was 6.75 cm (2.5-14 cm). Intrahepatic CC was located in the right liver lobe (n = 8) or in the left liver lobe (n = 6). Underlying liver disease was found in 42.9% (n = 6) of patients with IHCC. Five of 14 patients with IHCC underwent major hepatectomy (Table 2).

Postoperative outcomes are summarized in Table 3. The mortality rate was 8.5% (n = 4) after resection. The causes of death were PHLF (n = 2), pulmonary embolism (n = 1, 6 PODs), and myocardial infarction (n = 1, 22 PODs). Post-hepatectomy liver failure was observed in 2 of 3 patients who underwent PVR. Portal vein thrombosis (PVT) was determined in both of these cases. Portal vein flow was re-established after intravascular recombinant tissue plasminogen activator (tPA) infusion and placement of an endovascular stent via a percutaneous route in 1 patient. The patient rapidly improved from grade B PHLF. The other patient with PVT did not respond to endovascular treatment and died in 4 PODs. Post-hepatectomy liver failure was observed in 2 other patients due to borderline remnant liver volume (about 40%) after major hepatectomy. Both patients were classified as grade C PHLF. One of them survived with the help of our algorithm to support the failing liver; the other patient died in 3 PODs. Postoperative complications occurred in 68.1% of patients. Ten of 18 patients with surgical site infection (SSI) suffered from organ/space SSI. Biliary leak was detected in 7 of 10 patients with organ/space SSI. The majority of patients (n = 9/10) recovered with percutaneous drainage of the collection and antibiotic treatment. Re-laparotomy was required for a patient with anastomotic leak from jejuno-jejunostomy. In the histopathological evaluation, the R0 resection rate in patients with PHCC was 73% (Table 2).

**Figure 2.**
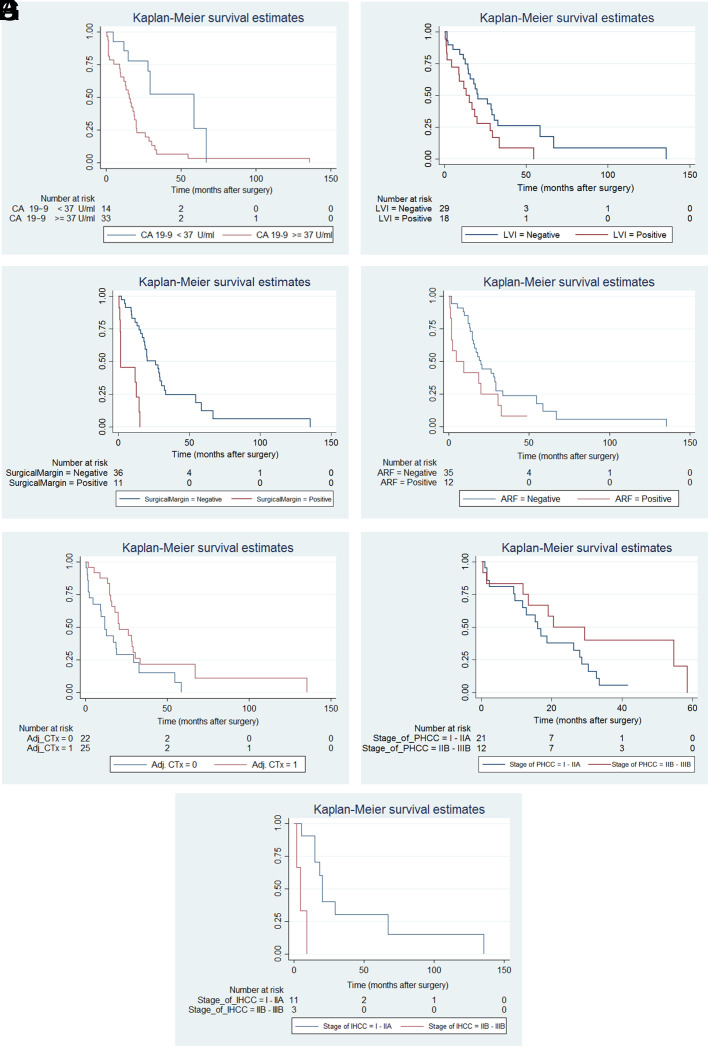
Effects of CA19-9 levels on survival (A), survival of patients with or without lymphovascular invasion (LVI) (B), the effects of surgical margin status on survival (C), the effects of the development of postoperative acute renal failure (ARF) on survival (D), the effects of adjuvant chemotherapy (Adj CTx) on survival (E), survival of patients who underwent resection for perihilar cholangiocarcinoma (PHCC) according to TNM staging (F), and survival of patients who underwent resection for intrahepatic cholangiocarcinoma (IHCC) according to TNM staging (G).

The mean OS time of PHCC cases that underwent R0 resection was 26.5 months (±24.8). The mean OS time of patients who underwent resection for IHCC was 28.7 months (±35.5) (Table 3). The univariate analysis indicated that OS was poorly affected by high CA19-9 levels (≥37 U/mL) (Figure 2A), the presence of lymphovascular invasion (Figure 2B), positive surgical margins after resection (Figure 2C) and the development of postoperative acute renal failure (Figure 2D) (Table 4). The OS of patients receiving adjuvant chemotherapy was significantly longer than that of patients who did not receive it (*P* = 0.071) (Figure 2E). Survival curves of patients who underwent resection for PHCC and IHCC according to AJCC staging are shown in Figure 2F and Figure 2G, respectively. The multivariate analyses indicated that CA19-9 levels more than 37 U/mL and positive surgical margins after resection were independent factors for poor OS after surgical treatment of CC (Table 4).

## Discussion

The present study showed that surgical treatment for PHCC and IHCC could be safely performed without portal vein embolization (PVE). The incidence of clinically relevant complications was comparable with the literature. The importance of positive surgical margins, high CA 19-9 levels, lymphovascular invasion, and postoperative renal failure on the poor prognosis is also verified. The OS time of patients who underwent R0 resection for PHCC or IHCC was found to be comparable with the literature.

The existence of a malignant featured stricture in the liver hilum in CT or magnetic resonance imaging series is generally considered as a basis for further diagnostic work-up for PHCC.^1^ According to the high false-negative rate of preoperative biopsy or intraluminal brushing, we did not perform a preoperative biopsy. As indicated by *Cillo*, it is critical to perform all imaging studies before biliary drainage.^1^ In contrast to a previous report by Corvera et al^15^ including 15% benign pathology in the resected specimens, pathological examination revealed malignant pathology in all of our patients preoperatively diagnosed as PHCC.

The clearest indication for preoperative biliary drainage (PBD) is obstructive cholangitis.^1^ In the absence of cholangitis, PBD of the remnant liver is debated. However, obstructive jaundice is associated with a pro-inflammatory state, and most of the surgeons used to perform PBD in jaundiced patients with PHCC before liver resection to decrease bleeding and postoperative complications.^1,3^ Despite the absence of large RCTs for the selection of PTBD, endoscopic biliary drainage, or endoscopic nasobiliary drainage for PBD, PTBD is an advantageous method for determining the determination of proximal extent of the tumor in the intrahepatic bile ducts. Insufficient remnant liver volume and function pose a risk for PHLF in the patient. Experienced Japanese centers recommend PVE for remnant liver volume below 40%.^3,16^ This approach resulted in a PHLF rate of 3.2% and a postoperative mortality rate of 1.4%.^1^ In our series, PVE was not perform to increase liver remnant volume. Therefore, our PHLF rate was higher than the Japanese series.

Systematic meta-analyses have demonstrated that intermittent PTC and low-CVP surgery, along with a shorter hospital stay and operation time, minimize blood loss and the requirement for transfusion during liver resection.^17^ However, detailed dissection of the structures in the hilum of the liver, resection of the common bile duct, and major liver resection for PHCC increase the operative time compared to our general practice in liver resections.^8^ Treatment for PHCC involves lymphadenectomy of loco-regional lymph nodes in the hepatoduodenal ligament; however, this procedure mostly affects staging rather than survival.^1^ PVR and reconstruction may be essential, and they may help patients with PHCC survive longer and achieve higher resection rates and R0 resection rates.^1,16^ There have also been reports of hepatic artery reconstruction and excision used as surgical treatments for PHCC, which result in high rates of morbidity and mortality.^1,16^

Similar to the literature, 14.2% of our patients who underwent liver resection for IHCC initially presented with obstructive jaundice.^1^ In our series, the majority of IHCC patients do not have any underlying liver disease. IHCC and HCC may be difficult to distinguish in imaging examinations.^1^ In our study group, synchronous occurrence of both tumors was also determined in a 48-year-old male patient with chronic hepatitis B virus infection.^18^ Although both CCs and HCCs have been identified as originating from distinct stem cell niches, the latest World Health Organization classification of the digestive system now recognizes the entity of combined hepatocellular-cholangiocarcinomas.^19^ Tumor markers in the serum have a limited role in the diagnostic evaluation for IHCC. The risk of recurrent disease along the biopsy tract and difficulty in pathological discrimination of IHCC from metastatic disease are debating issues in the preoperative biopsy for IHCC.^1^ Despite the guidelines recommending lymphadenectomy in patients with resectable IHCC,^20^ we did not perform routine lymphadenectomy before 2015. To eliminate some optimization problems for our patients according to the 8th edition of the AJCC staging system for IHCC, we used the 7th edition of the AJCC staging system for IHCC in our study group. On the other hand, lymphadenectomy seems to be mostly a staging operation with minimal impact on OS.^1^

The amount of bleeding in the PHCC and IHCC surgeries was discovered to be less than our bleeding threshold, which is used to calculate the morbidity associated with liver surgery.^8^ Grade-3 complication rate in our study is found comparable with the recent multicenter analysis.^21^ The rate of pulmonary complications in this group of patients was found higher than our general practice in liver surgery.^6-9^ The incidence of biliary leaks in this study appears comparable with the recent multicenter studies.^21^ The overall rate of PHLF in our study group is also comparable with that in the literature.^1,3,17,21^ With our aggressive treatment policy,^12,22^ patients suffering from PHLF had a high survival rate. Important factors contributing to the positive recovery rates associated with PHLF include aggressive measures to address vascular abnormalities due to surgery and the prudent use of non-biological liver support in the therapy algorithm against liver failure. The 30-day mortality rate in our study group is also comparable with the experienced centers in the field.^1,21^

Independent roles of the positive surgical margin and high CA19-9 levels on the poor prognosis are verified in this study. The adverse effects of postoperative renal failure and lymphovascular invasion, and the favorable effect of adjuvant chemotherapy on the prognosis are also determined in the univariate analysis. The best outcomes are observed in centers with high patient volumes. Yet, recurrence of disease is observed in 80% of patients, mostly within the first 2 years following surgery.^1,21^ The OS period of our patients who underwent R0 resection for PHCC was found to be lower than that of experienced centers. Limited administration of adjuvant chemotherapy or adjuvant chemo-radiotherapy may be the cause of the low OS period found in this study. The OS period of our patients undergoing resection for IHCC was found to be comparable with the literature.^1^ The high rates of adjuvant chemotherapy or chemo-radiotherapy administration and effective treatment of recurrences with re-resection and loco-regional treatments in this study may have positively affected the OS period in IHCC patients.

The main limitation of this study is the small sample size. It may preclude powerful analysis of prognostic factors. This study, however, presents a distinct and comparable range of patients from a university hospital in the northeastern region of Türkiye.

In conclusion, surgical resection is the mainstay of treatment for PHCC and IHCC. Our research pointed out the prognostic significance of CA 19-9 level, surgical margins after resection, lymphovascular invasion, postoperative renal failure, and adjuvant chemotherapy among PHCC and IHCC patients. In advanced stages of IHCC, the combination of loco-regional therapies and repeat surgery, along with the enhanced efficacy of systemic chemotherapy, plays a significant role in a patient’s survival. We believe that this study will significantly advance efforts to increase patient survival in cases of PHCC and IHCC.

## Figures and Tables

**Table 1.** Patient’s Characteristics

Variable	N = 117
Age (median, min-max)	67 (33-100)
Gender (%)	
Male	66 (56.41)
Female	51 (43.59)
BMI (median, min-max)	26.37 (18-40.2)
Comorbidities (%)	
Presence of anemia	57 (48.7)
Diabetes	24 (20.5)
Systemic arterial hypertension	39 (33.3)
Coronary artery disease	22 (18.8)
Chronic pulmonary disease	10 (8.5)
Primary sclerosing cholangitis	2 (1.7)
Smoking (%)	29 (24.7)
ASA Score (%)	
I-II	74 (63.2)
III-IV	43 (36.7)
Chronic HBV infection (%)	3 (2.56)
Chronic HCV infection (%)	3 (2.56)
Presence of cirrhosis (%)	9 (7.64)
Total bilirubin at admission (%)	
≥3 mg/dL	80 (68.36)
<3 mg/dL	37 (31.62)
Preoperative CEA level (%)	
≥5 U/mL	53 (45.3)
<5 U/mL	64 (57.7)
Preoperative CA19-9 level (%)	
≥37 U/mL	93 (79.5)
<37 U/mL	24 (20.5)
PHCC (%)	91 (77.8)
Bismuth–Corlette type	
Type I	20 (21.9)
Type II	12 (13.2)
Type IIIA	15 (16.4)
Type IIIB	26 (28.5)
Type IV	18 (19.8)
IHCC (%)	26 (22.2)
Patients considered inoperable or unresectable (%)	70 (59.8)
PHCC	58 (82.8)
Bismuth–Corlette type	
Type I	15 (26.1)
Type II	11 (18.9)
Type IIIA	8 (13.7)
Type IIIB	11 (18.9)
Type IV	13 (22.4)
IHCC	12 (17.1)

ASA, American Society of Anesthesia; BMI, Body mass index; CA, Carbohydrate antigen; CEA, Carcinoembryonic antigen; HBV, Hepatitis B virus; HCV, Hepatitis C virus; IHCC, intrahepatic cholangiocarcinoma; PHCC, perihilar cholangiocarcinoma.

**Table 2.** Demographic, Perioperative and Pathological Parameters of the Patients Who Underwent Liver Resection

Variable	N = 47
Age (mean, ±SD)	62.32 (±4.41)
Gender (%)	
Male	30 (63.8)
Female	17 (36.2)
BMI (mean, ±SD)	27.08 (±4.41)
Comorbidities (%)	
Diabetes	11 (23.4)
Systemic arterial hypertension	11 (23.4)
Coronary artery disease	6 (12.8)
Chronic pulmonary disease	5 (10.6)
Primary sclerosing cholangitis	1 (2.1)
ASA Score (%)	
I-II	36 (76.6)
III-IV	11 (23.4)
Chronic HBV infection (%)	2 (4.3)
Chronic HCV infection (%)	2 (4.3)
Presence of cirrhosis (%)	4 (8.5)
Preoperative biliary drainage (%)	34 (72.3)
PHCC	32 (97)
IHCC	2 (14.3)
Major hepatectomy (%)	33 (70.2)
Right hepatectomy (with BDR/without BDR)	9 (27.3)
Extended right hepatectomy (with BDR/without BDR)	2 (6.1)
Left hepatectomy (with BDR/without BDR)	17 (51.5)
Extended left hepatectomy (with BDR/without BDR)	4 (12.1)
Left lateral sectorectomy, segment 5 and 7 resection	1 (3)
Minor hepatectomy (%)	9 (19.2)
Sectorectomy	3 (33.3)
Posterior sectorectomy	1 (33.3)
Lateral sectorectomy	2 (66.7)
Segmentectomy	8 (88.9)
Segment 4B	2 (25)
Segment 5	3 (37.5)
Segment 6	2 (25)
Segment 7	1 (12.5)
BDR alone (%)	5 (10.6)
Portal vein resection (%)	3 (6)
Portal triad clamping period (minutes, mean ±SD)	18 (±16.9)
Operative time (minutes, mean ±SD)	211 (±117)
Intraoperative bleeding amount (milliliters, median, min–max.)	250 (10-3500)
R0 resection for PHCC (%)	24 (73)
R1 resection for PHCC (%)	7 (21)
R2 resection for PHCC (%)	2 (6)
TNM Staging of cases with PHCC (%)	33
I	3 (9.1)
II	18 (54.5)
III	12 (36.4)
IV	0
R0 resection for IHCC (%)	14 (100)
TNM Staging of cases with IHCC (%)	14
I	9 (64)
II	2 (15)
III	3 (21)
IV	0
Histopathological type of tumor (%)	
Adenocarcinoma	44 (94)
Mucinous adenocarcinoma	1 (2)
Neuroendocrine tumor	2 (4)
Tumor differentiation (%)	
Well	26 (56)
Moderately	20 (42)
Poorly	1 (2)
Presence of perineural invasion (%)	28 (59.6)
Presence of peritumoral fibrosis (%)	29 (61.7)
Mild	5 (17.2)
Moderate	14 (48.3)
Severe	10 (34.5)
Presence of tumor necrosis (%)	8 (28.5)

ASA, American Society of Anesthesia; BDR, bile duct resection; BMI: body mass index; HBV, hepatitis B virus; HCV: hepatitis C virus; IHCC, intrahepatic cholangiocarcinoma;PHCC, perihilar cholangiocarcinoma; TNM, extent of the tumor-spread of the lymph nodes-presence of metastasis.

**Table 3.** Postoperative Parameters of the Patients Who Underwent Liver Resection

Variable	N = 47
Morbidity (%)	32 (68.1)
Type of complication (severity of complication–Dindo grade or ISGLS staging) (%)	
Pulmonary complications	21(44.7)
Atelectasis	15 (31.9)
Dindo 1	9 (60)
Dindo 2	6 (40)
Pleural effusion	17 (36.2)
Dindo 1	6 (35.3)
Dindo 2	3 (17.6)
Dindo 3a	8 (47.1)
Pneumonia	3 (6.4)
Dindo 2	3 (100)
Pulmonary embolism	1 (2)
Dindo 5	1 (100)
Wound infection	18 (38.3)
Dindo 1	1 (6)
Dindo 2	7 (38)
Dindo 3a	9 (50)
Dindo 3b	1 (6)
Biliary leak	7 (14.9)
Dindo 2	1 (14.3)
Dindo 3a	5 (71.4)
Dindo 3b	1 (14.3)
ARF	10 (21.3)
Dindo 1	4 (40)
Dindo 2	6 (60)
Posthepatectomy liver failure	4 (8.5)
GRADE B (ISGLS)	1 (25)
GRADE C (ISGLS)	3 (75)
Relaparotomy (%)	1 (2.1)
30 days mortality (%)	4 (8.5)
Requirement of ICU care (%)	7 (15)
Length of postoperative hospital stay (mean ± SD)	28 (±23)
Adjuvant chemotherapy for PHCC (%)	5 (15.2)
R0	4 (16.7)
R2	1 (50)
Adjuvant chemo-radiotherapy for PHCC (%)	8 (24.2)
R0	6 (25)
R1	2 (28.6)
Adjuvant chemotherapy for IHCC (%)	7 (50)
Adjuvant chemo-radiotherapy for IHCC (%)	5 (35.7)
Re-resection for IHCC (%)	2 (14.3)
TACE for IHCC (%)	2 (14.3)
SIRT for IHCC (%)	1 (7.1)
Median follow-up period for PHCC (month, min-max)	15.6 (0.1-58.6)
DFS period in patients with PHCC (month, mean ± SD)	14 (13.4)
OS period in patients with R0 resection for PHCC (month, mean ± SD)	26.5 (24.8)
OS period in patients with R1 resection for PHCC (month, mean ± SD)	5.49 (5.5)
Median follow-up period for IHCC (month, min-max)	15.6 (1.6-135.5)
DFS period in patients with IHCC (month, mean ± SD)	18.4 (35)
OS period in patients who underwent liver resection for IHCC (month, mean ± SD)	28.7 (35.5)

ARF, acute renal failure; DFS, disease-free survival; ICU, intensive care unit; IHCC, intrahepatic cholangiocarcinoma; ISGLS, International Study Group of Liver Surgery; OS, overall survival; PHCC, perihilar cholangiocarcinoma; SIRT, selective internal radiation therapy; TACE, transarterial chemoembolization.

**Table 4.** Significant Factors for Survival by Univariate and Multivariate Analyses of Patients Who Underwent Resection for Cholangiocarcinoma

Variable	N	Univariate		Multivariate	
		HR (95% CI)	*P*	HR (95% CI)	*P*
Age					
<65 yr	25	1.49 (0.78-2.83)	.224		
≥65 yr	22				
Sex					
Male	30	1.74 (0.88-3.45)	.101		
Female	17				
Preoperative CA19-9					
<37 U/mL	14	3.12 (1.41-6.90)	.005	2.57 (1.12-5.90)	.027
≥37 U/mL	33				
Postoperative ARF					
No	35	1.89 (0.92-3.88)	.0781		
Yes	12				
Lymphovascular invasion					
No	29	1.93 (1.002-3.73)	.049		
Yes	18				
Perineural invasion					
No	19	1.27 (0.66-2.46)	.470		
Yes	28				
Tumor differentiation					
Well	26	1.37 (0.72-2.61)	.340		
Moderate/Poor	21				
Peritumoral fibrosis					
Mild/Moderate	19	1.03 (0.55-1.92)	.932		
Severe	10				
Tumor necrosis					
No	20	1.45 (0.55-3.84)	.458		
Yes	8				
Positive surgical margin					
No	36	8.25 (3.24-20.99)	<.001	6.14 (2.34-16.11)	<.001
Yes	11				
Adjuvant chemotherapy					
No	22	0.55 (0.29-1.05)	.071		
Yes	25				

ARF, acute renal failure; CA, carbohydrate antigen; HR, hazard ratio.

## Data Availability

The data that support the findings of this study are available on request from the corresponding author.
